# *Begonia shenzhenensis*, a new species of Begoniaceae from Guangdong, China

**DOI:** 10.3897/phytokeys.178.66462

**Published:** 2021-06-04

**Authors:** Dai-Ke Tian, Bin Chen, Yan Xiao, Min-Min Zheng, Bin-Jie Ge

**Affiliations:** 1 Shanghai Chenshan Plant Science Research Center, Chinese Academy of Sciences, 3888 Chenhua Road, Songjiang, Shanghai 201602, China Shanghai Chenshan Plant Science Research Center, Chinese Academy of Sciences Shanghai China; 2 Shanghai Key Laboratory for Plant Functional Genomics and Resources, Shanghai Chenshan Botanical Garden, 3888 Chenhua Road, Songjiang, Shanghai 201602, China Shanghai Chenshan Botanical Garden Shanghai China; 3 Eastern China Conservation Center for Wild Endangered Plant Resources, Shanghai 201602, China Eastern China Conservation Center for Wild Endangered Plant Resources Shanghai China; 4 The University of Chinese Academy of Sciences, Beijing 100049, China The University of Chinese Academy of Sciences Beijing China

**Keywords:** Conservation status, deciduous, morphology, rhizomatous begonia, southern China, taxonomy

## Abstract

*Begonia shenzhenensis* D.K.Tian & X.Yun Wang, **sp. nov.**, a new species in Begonia sect. Platycentrum of Begoniaceae from Shenzhen of Guangdong province, China, is described and illustrated. Morphologically, it is primarily similar to *B. coelocentroides* in the same section but differs by its denser hairs on leaf, petiole, and pedicel, abtuse anther apex, hairy ovary, and narrower adaxial fruit wing. Based on only one small population found to date, its conservation status is assigned to Critical Endangered according to the IUCN Red List Categories and Criteria.

## Introduction

Guangdong province is located in southern China, and it borders Macau and Hong Kong, two Chinese special administrative regions. Numerous surveys on plant diversity of this province have been widely conducted by a number of botanists, mainly from several research institutions, including South China Botanical Garden of Chinese Academy of Sciences, Sun Yat-sen University, Guangdong Academy of Forestry, South China Agricultural University, and others. Nonetheless, some new species and new records have been recently discovered (Huang et al. 2020; Zhou et al. 2020), including two begonia species (Ding et al. 2018; Tu et al. 2020). Here we describe and illustrate a new species of *Begonia*, *Begonia shenzhenensis* D.K.Tian & X.Yun Wang, which increases the number of begonias species to 14 in Guangdong province. The other 13 species are *B. circumlobata* Hance, *B. coptidifolia* H.G.Ye, F.G.Wang, Y.S.Ye & C.-I Peng, *B. cucullata* Willd. (naturalized), *B. edulis* H.Lév., *B. ehuangzhangensis* Q.L.Ding, W.Y.Zhao & W.B.Liao, *B. fimbristipula* Hance, *B. fordei* Irmsch., *B. grandis* Dryand., *B. guangdongensis* Wen-Hui Tu, Bing-Mou Wang & Yu-Ling Li, *B. handelii* Irmsch., *B. leprosa* Hance, *B. longifolia* Blume, and *B. palmata* D.Don.

## Taxonomic treatment

### Begonia shenzhenensis

Taxon classificationPlantaeCucurbitalesBegoniaceae

D.K.Tian & X.Yun Wang sp. nov.

50D8D710-BB36-5556-ABAE-4B2BC3CCA3EB

urn:lsid:ipni.org:names:77217426-1

**Figure 1.**
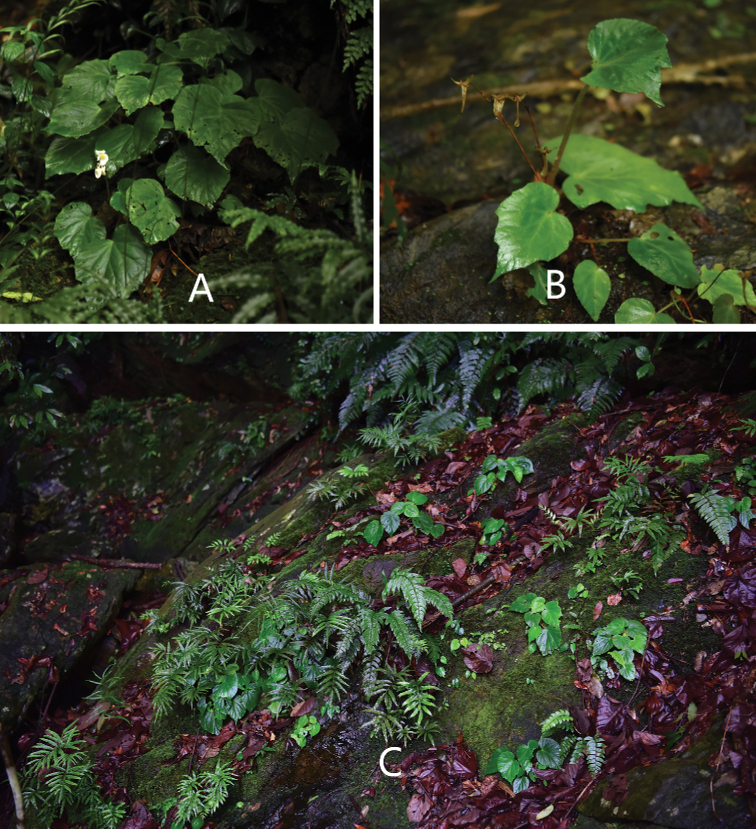
Plants and habitat of *B. shenzhenensis* D.K.Tian & X.Yun Wang **A** plant at the earliest flowering time (6 June 2020) **B** plants with the dried fruits matured in the previous year **C** habitat showing mature plants and seedlings of the new species growing together with mosses and ferns above the rocks. (Photo by Dai-Ke Tian).

**Figure 2.**
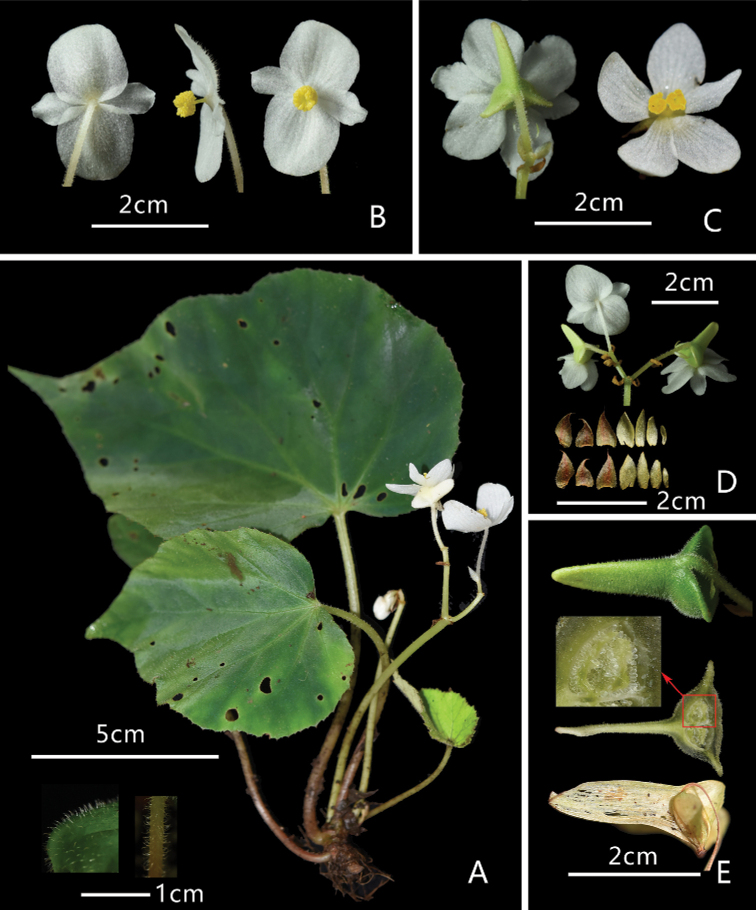
Morphology of *Begonia shenzhenensis* D.K.Tian & X.Yun Wang **A** one of the largest plants with flowers (left corner: sections of leaf and petiole showing hairs) **B** staminate flowers showing in different directions **C** pistillate flowers, bracts (left: back view of 6-tepalled flower; right: front view of 5-tepalled flower) **D** inflorescence, stipules (left) and bracts (right) **E** fruits (top: fresh, bottom: dried) and ovary dissection (Photo by Dai-Ke Tian).

Chinese name: 深圳秋海棠 Figs 1 , 2

#### Type.

**China**. Guangdong: Shenzhen (深圳), Pingshan (坪山) District, Tiantou Mountain (田头山) Natural Reserve, on shallow humus soil above the rocky surface under the forest along streams, 22°43'29"N, 114°24'2"E, elev. 70 m, 6 June 2020, Dai-Ke Tian, Xiao-Yun Wang, Bin Chen & Shi-Ping Zhong, TDK4160 (holotype, CSH! CSH0182483).

#### Specimen examined.

Same locality as type, 21 September 2019, Xiao-Yun Wang, TDK3981 (paratype, CSH); Shanghai Chenshan Plant Science Research Center, cultivated plants under 25 °C room temperature, 7 November 2020, Dai-Ke Tian, TDK4832 (paratype, CSH).

#### Diagnosis.

The new species is most similar to *B. coelocentroides* Y.M.Shui & Z.D.Wei in the same section, Begonia Section Platycentrum (J.F.Klotzseh) A.DC. with rhizomatous habit, four tepals of staminate flowers, five tepals of pistillate flower and two-localed ovary, but clearly differs by its densely (vs. sparsely) hairy petioles and blades, hairy (vs. glabrous) ovaries, abtuse (vs. concave) anther apex, and narrower (vs. wider) adaxial fruit wing. It is also close to the small-sized individuals (before mature) of *B. edulis* in appearance but differs by its short and small (vs. tall and large) and non-stemmed (vs. stemmed) plants, and hairy (vs. glabrous) pedicels, flowers, and fruits (Table 1, Fig. 2).

**Table 1.** Comparison of *Begonia shenzhenensis* and *B. coelocentroides*.

	*B. shenzhenensis*	*B. coelocentroides*
Plant height	7–15 cm	5–10 cm
Leaf-blade	densely hairy	sparsely pilose to nearly glabrous
Petiole’s hair	dense	sparse
Peduncle and pedicel	hairy	glabrous
Outer staminate tepal	hairy	sparsely hairy
Anther apex	obtuse	concave
Fruit	densely hairy	glabrous
Adaxial wing	narrower, 8–10 mm wide	wider, 9–14 mm wide

***Herb*** perennial, rhizomatous, 7–15 cm tall, monoecious, epiphytic on rock with moss or humus soil. ***Rhizome*** creeping, 5–10 cm long, internodes unclear to 10 mm long, 5–13 mm thick, unbranched to rarely branched, greenish-brown. Aerial stem absent, or occasionally one very short internoded stem at anthesis. ***Leaves*** simple, basal, alternate, 4–8 each plant, deciduous in winter. ***Stipules*** persistent, pale pink, long triangular, 5–8 × 4–6 mm, abaxially hairy, apex with short arista. ***Petioles*** light-green to pink, 4–18 cm long, 1.5–5 mm thick, grooved in full-length, short greyish pubescent, less than 1.5 mm long, hair tips often curly. ***Leaf-blades*** obliquely oval-cordate, 5–22 × 3.5–16 cm; adaxially green, rarely dark green, rough, short greyish strigose, less than 1 mm long; adaxial veins slightly convex at the base, concave upper part; abaxially greyish-green or lightly red-purple, veins convex, with short greyish strigose, 1–1.5 mm long, leaf veins 7 to 6; ***Leaf base*** shallowly cordate, obtuse to nearly overlapping, decurrent part 1–4 cm long; leaf margins serrate, rarely double serrate, occasionally cleft-teethed, with arista; apex acuminate or short-caudate. ***Inflorescences*** 2–4, successively growing from leaf axil of the stem near the shoot, 11–17 cm long; peduncle light green, 8–15 cm long, 2–3.5 mm thick, greyish-white villose, pedicel less hairy, each inflorescence 3–7 flowers, 0–2 pistillate. ***Bracts*** and bracteoles greenish-white, long triangular, bracts 5–12 × 3–7 mm; bracteoles 4–11 × 2–6 mm, both abaxially sparsely white pubescent, ca. 1 mm long, apex acuminate, with sparse cilia. ***Staminate flower***: pedicel white or light-green at low part, 1.8–3.4 (4.5) cm long, 1–2 mm thick, short greyish white-pubescent (less than 1 mm long); flower white, 2.5–4.5 × 2.4–3.7 cm; outer 2, ovate, entire, 1.2–2.2 × 1.2–2.1 cm, central part thick and abaxial with short grayish white or pink pubescent, indumentum same as pedicel; inner 2, long obovate–lanceolate, glabrous, 10–19 × 5–9 mm. androecium, 3–4 × 4–5 mm, stamens 50–92, filaments basally connate into stamen column, 2–3 mm × ca. 1 mm, free part 1–2 mm long, anthers yellow, 0.8–1.2 mm long. ***Pistillate flower***: a pair of pale green bracteoles persistent near ovary, 3–10 × 1–4 mm; pedicel 9–18 mm long, 1.2–2 mm thick, with extremely short greyish white pubescent, less than 0.5 mm long; flower 30–37 × 25–37 mm, tepals 5 (rarely 6), outer 4 (5), slightly large, 13–18 × 7–15 mm, sparsely greyish white short pubescent, inner 1, small, glabrous, oblanceolate, 11–15 × 5–9 mm wide; gynoecium 3–4 × 3–7 mm, styles 2, stigmas 2, nearly U-shape branched, both sides spirally twisted nearly one circle; ovary greenish-white, short hairy, placentation axile, 2-loculed, each placenta 2–branched; ***Peduncles*** light-green, hairy, 15–32 cm long, ca 2 mm thick, hairy, a pair of green bracteoles often persistent near ovary; ***Fruits*** capsule, green, densely short grayish-white pubescent, 6–10 × 5–8 mm, 3–winged, unequal, abaxial wing long rectangular or subtriangular, 18–28 × 8–10 mm; lateral wings narrow, sickle-shaped or cornice or auriculate triangular, ca. 4–8 × 8–10 mm. Flowering June–July. Fruiting July–October.

#### Etymology.

The specific epithet refers to the name of Shenzhen, which includes the type locality of the new species.

#### Distribution and habitat.

**Figure 3.**
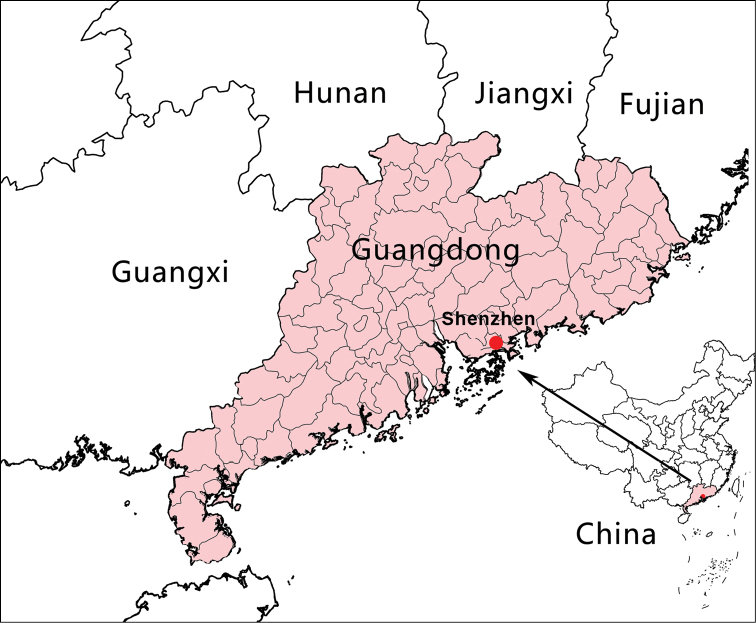
Distribution of *Begonia shenzhenensis* (red dot) from Shenzhen, Guangdong of China.

The new species is only known from its type locality in Tiantou Mountain Natural Reserve of Pingshan District of Shenzhen, Guangdong Province, China (Fig. 3). It grows together mainly with mosses, ferns, and other herbs on shallow humus soil above the rocks along a small stream under forest canopy.

#### Conservation status.

*Begonia shenzhenensis* is only observed in its type locality with a very small population containing fewer than 50 individuals. Due to the plant’s relatively low ornamental value, it is possibly unlikely to be collected by plant hunters. However, there are no suitable places nearby for spreading its population. Therefore, this species should be considered as Critical Endangered (CE) according to the IUCN Red List Categories and Criteria (IUCN 2019) based on the current data.

#### Note.

The new species was first disovered by Mr. Xiao-Yun Wang, a plant enthusiast working at Hechang Weilai Science and Technology (Shenzhen) Co. Ltd. in Guangdong province of China. On 21 September 2019, Xiao-Yun Wang posted photos of a wild begonia from Shenzhen of Guangdong province, China. At the request of Dr. Dai-Ke Tian, the living plants were collected and introduced to Shanghai Chenshan Botanical Garden for further study. The introduced plants grow well in two plastic pots placed in a squared polymethyl box at 25 °C room temperature and bloom from November to June. On 6 June of 2020, with the help of Xiao-Yun Wang, Dr. Dai-Ke Tian and Dr. Bin Chen conducted a field survey on this species and further confirmed that it is new to science.

## Supplementary Material

XML Treatment for Begonia shenzhenensis

## References

[B1] Ding QL Zhao WY Yin QY Ye HG Song HZ Liao WB (2018) *Begonia ehuangzhangensis* (sect. Diploclinium, Begoniaceae), a new species from Guangdong, China. Phytotaxa 381(1): 107–115. 10.11646/phytotaxa.381.1.14

[B2] Huang CY Meng KK Guo JQ Chen F Liao WB Fan Q (2020) *Primulina huangjiniana* (Gesneriaceae), a new species from Guangdong, China. Guihaia 10: 1429–1437.

[B3] IUCN (2019) Guidelines for using the IUCN Red List Categories and Criteria. Version 14. Prepared by the Standards and Petitions Subcommittee. https://www.iucnredlist.org/resources/redlistguidelines [accessed on 15 March 2021]

[B4] Tu WH Wang BM Yao G Huang JX Li YL (2020) *Begonia guangdongensis*, a new species of *Begonia* (Begoniaceae) from Guangdong, China. PhytoKeys 162: 29–36. 10.3897/phytokeys.162.51913 33117069 PMC7560510

[B5] Zhou W Song CF Wu BC (2020) New records of Apiaceae distributed in Guangdong and Guangxi. Journal of Plant Resources and Environment 29(2): 78–80.

